# Hemopericardium and Cardiac Tamponade Secondary to Migrated Inferior Vena Cava Filter

**DOI:** 10.1155/2018/5919767

**Published:** 2018-11-21

**Authors:** Jeet J. Mehta, Benjamin DeMarco, John P. Vavalle, Khola S. Tahir, Joseph S. Rossi

**Affiliations:** ^1^Internal Medicine/Pediatrics, University of Kansas School of Medicine-Wichita, USA; ^2^Department of Internal Medicine, University of North Carolina Hospitals & Clinics, Chapel Hill, NC, USA; ^3^Department of Cardiology, University of North Carolina Hospitals & Clinics, Chapel Hill, NC, USA

## Abstract

A 73-year-old female presented with cardiogenic shock secondary to hemopericardium and cardiac tamponade. Imaging revealed two fractured legs of an inferior vena cava filter, with one leg within the anterior myocardium of the right ventricle and another penetrating the inferior septum through the middle cardiac vein. Hemopericardium and cardiac tamponade were treated with pericardiocentesis. A multidisciplinary meeting resulted in deferring further action against the embedded fractured legs of the filter with consideration of the patient's age and comorbidities. This case report should alert clinicians to think about hemopericardium as a cause of cardiac tamponade and cardiogenic shock in a patient with a history of an inferior vena cava filter placement.

## 1. Introduction

Inferior vena cava (IVC) filters are commonly placed in an effort to prevent pulmonary embolism. The only accepted indication for placement of a vena cava filter is for patients who have venous thromboembolism and an absolute contraindication to anticoagulation [1]. There are important, well-known complications of filter placement, such as bleeding, infection, and access site complications including hematoma or arteriovenous fistula. Long-term complications include filter protrusion resulting in perforation of the IVC or surrounding organs and filter fracture and/or embolization. There is limited data regarding the safety and efficacy of the various filter types. The PRESERVE (predicting the safety and effectiveness of inferior vena cava filters) trial was initiated in 2014 to evaluate the safety and efficacy of six different IVC filters over long term.

We present a case of an elderly female who was admitted from a nursing home for acute seizure activity and acute hemodynamic instability found to be secondary to hemopericardium resulting in cardiac tamponade.

## 2. Case Presentation

A 73-year-old female with a past medical history of chronic pancreatitis, type 2 diabetes mellitus, gastroesophageal reflux disease, lower gastrointestinal (GI) bleed, hypertension, paroxysmal atrial fibrillation, cerebrovascular accident, seizure disorder, and pulmonary embolism (PE) presented to an outside hospital after a reported seizure at her nursing home. Emergency medical services were called at the nursing home and the patient was reportedly hypoxic with oxygen saturation in the 70s on room air with subsequent development of agitation and lethargy after the seizure. The patient was transferred to an outside hospital's emergency department (ED), where additional tonic-clonic activity was noted. She subsequently developed hypotension with a blood pressure of 52/36 mmHg that was refractory to crystalloid intravenous fluid resuscitation. Central venous catheter (CVC) was placed and norepinephrine was started for persistent hypotension. Labs were notable for leukocytosis, troponin elevation, and low mixed venous saturation on CVC venous blood gas. The patient was subsequently transferred to a tertiary care facility cardiac intensive care unit for evaluation of cardiogenic shock.

Upon arrival, the patient was alert and following commands, but disoriented. She was still requiring supplemental oxygen and norepinephrine for hypotension. Given the concern for cardiogenic shock, a stat bedside transthoracic echocardiogram was obtained, which demonstrated a large pericardial effusion with tamponade physiology. The patient was urgently taken to the cardiac catheterization lab for pericardiocentesis with a drain placement. This yielded 580 ml of hemorrhagic fluid with rapid improvement in hemodynamics following drainage. During the pericardiocentesis, fluoroscopy demonstrated two embolized fragments of the IVC filter within the right ventricle (RV). Review of past imaging revealed that the embolized fragment was visualized on computer tomography (CT) of the chest one year and one month prior to presentation. Upon further investigation from family, it was discovered that the patient's Bard G2 retrievable IVC filter was placed in April 2007 in the context of a PE with concurrent GI bleed. Pericardial fluid culture was negative, and cytology and fluid analysis were consistent with hemopericardium as cause of the pericardial effusion.

**Figure 1:**
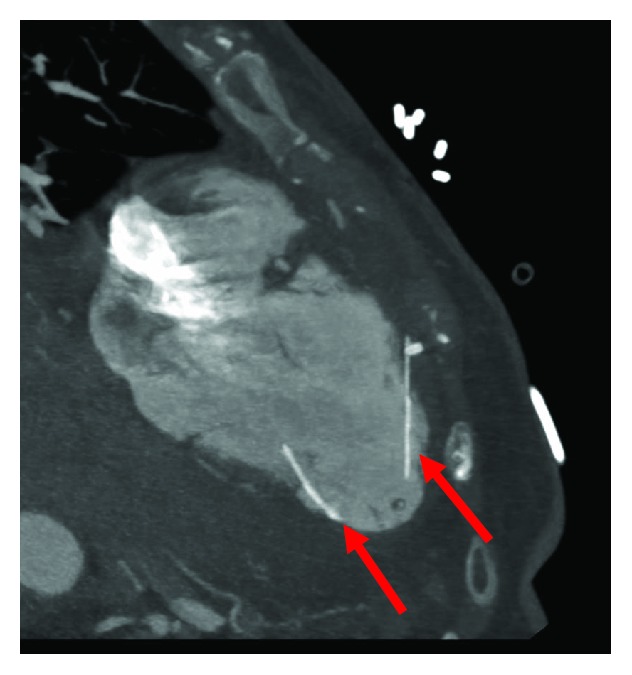
Fractured IVC filter leg embedded within the anterior myocardium and inferior septum of the RV (arrows).

**Figure 2:**
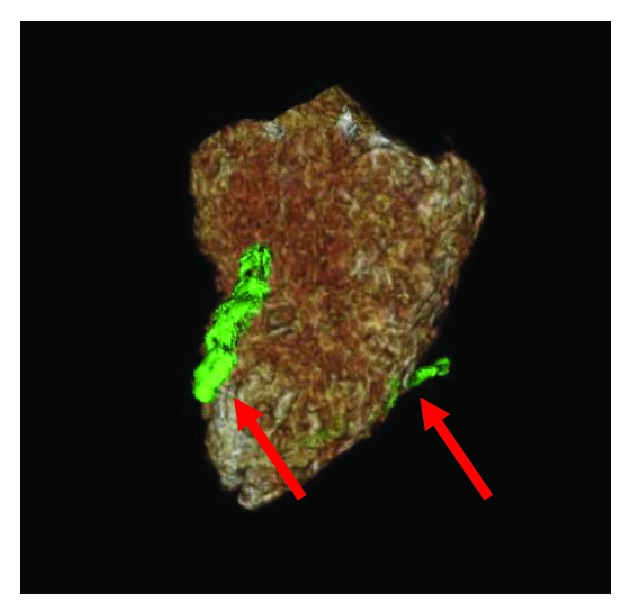
Fractured IVC filter leg embedded within the anterior myocardium and inferior septum of the RV (arrows).

Cardiothoracic surgery was consulted due to the embolized fractured IVC filter. Surgery recommended cardiac CT angiogram (CTA) for further visualization of the fragment. Cardiac CTA demonstrated two fractured legs of the IVC filter, with one leg within the anterior myocardium of the RV and another penetrating the inferior septum through the middle cardiac vein (Figure 1). 3-D reconstruction of the RV further visualized the two legs of the embolized IVC filter (Figure 2). Repeat transthoracic echocardiogram one day after the pericardiocentesis revealed no reaccumulation of the pericardial effusion and the pericardial drain was successfully removed. A multidisciplinary team discussion took place with the family who elected to defer surgical removal of the IVC filter unless pericardial effusion reaccumulated due to the patient's comorbidities and age. She was discharged to her assisted living facility, and continues to do well at the time of this report.

## 3. Discussion

Although IVC filters offer an effective means of preventing pulmonary embolism in a population where anticoagulation is contraindicated, despite improved filters and insertion techniques, complications still occur. The major risks associated with the placement of an IVC filter include pneumothorax, hemorrhage, filter misplacement, and vessel injury [2]. Migration and/or embolization of a fractured IVC filter remains one of the late rare, but potentially lethal complications. Filter migration has a reported incidence of 3% to 12%. Complete filter displacement with migration to the heart is even more rare, ranging from 0.1% to 1.2% of the cases, with a variable prevalence depending on the filter type [2, 3]. Several factors have been recognized for IVC filter dislodgement and fracture, including technical errors, equipment malfunction, mega cava or an increase in IVC diameter from Valsalva maneuvers, poor alignment of the filter with the IVC, J-wire entrapment during central line placement, and thrombus pushing the filter proximally [4, 5].

Filter fractures are more common with retrievable filters, with the highest to lowest incidence in the following order: Bard G2, Cook Celect, Bard Recovery, Bard Eclipse, Cook Günther, Bard Meridian, Bard G2X, Bard Option, and OptEase filter. The most common location of a fractured fragment is the IVC wall and pulmonary artery, followed by the spine and heart. The Bard Recovery, Bard G2, and Cook Celect have the highest incidence of embolized filter fragments to the heart [5]. Our patient's Bard G2 filter has a five-year fracture prevalence of 38% [6, 7]. Intravascular filter fragments can be removed safely with success rates that vary according to location. In a study by Trerotola and Stavropoulos, 90% (43 of 48) of filters were retrieved from the IVC, 50% (three of six) from the heart, and 71% (17 of 24) from the pulmonary arteries. Because of extravascular fragment location and/or retrieval failure, only 50% of the patients were recognized to be fragment free [8].

One of the major ways to prevent this lethal complication is by focusing on early retrieval of the filter once protection from PE is no longer needed. Other indications for removal include locally retained intravascular fragment (within the IVC) as it poses a risk for central embolization and/or thrombus formation and in any of the patients who are symptomatic on a case-by-case basis after carefully weighing the risk-benefit ratio. By 2010, the Food and Drug Administration (FDA) received 921 reports of adverse events with IVC filters: of these reports, 328 involved device migration, 146 involved embolizations (detachment of device components), 70 involved perforation of the IVC, and 56 involved filter fracture [9]. The Bard G2 filter was taken off the market in 2010. In 2013, the Bard Denali filter was approved by the FDA to replace the Recovery, G2, G2 Express, Eclipse, and Meridian filters in the hopes of having a better-designed and long-term performance filter with fewer complications [6].

In conclusion, the ideal IVC filter design is evolving and remains unknown. Based on current literature, the fragments from fractured filters, when intravascular, can be successfully removed by using percutaneous interventional techniques in a large percentage of patients, but the success rate varies according to fragment location. For the extravascular fragments, however, only approximately half of the patients are rendered fragment free and extraction should be discussed on a case-by-case basis. In each case, it is important to remember that timely follow-up and early retrieval of the filter will help prevent long-term complications. Until the PRESERVE trial provides long-term data on safety and efficacy of the IVC filters (ALN, Option Elite Retrievable, VenaTech LP, Cook Günther Tulip, Bard DENALI, and Cordis OptEase Retrievable vena cava filter), case reports like these should alert clinicians to think about hemopericardium as a cause of cardiac tamponade and cardiogenic shock in a patient with a history of an IVC filter placement.
